# Investigation of the Mechanism of Action of AMPs from Amphibians to Identify Bacterial Protein Targets for Therapeutic Applications

**DOI:** 10.3390/antibiotics13111076

**Published:** 2024-11-12

**Authors:** Carolina Canè, Lidia Tammaro, Angela Duilio, Angela Di Somma

**Affiliations:** 1 CEINGE Biotecnologie Avanzate Franco Salvatore, 80145 Naples, Italy; carolina.cane@unina.it (C.C.); tammarol@ceinge.unina.it (L.T.); 2 Department of Chemical Sciences, University of Naples “Federico II”, Via Cinthia 4, 80126 Napoli, Italy; angela.duilio@unina.it; 3 National Institute of Biostructures and Biosystems (INBB), Via dei Carpegna 19, 00165 Roma, Italy

**Keywords:** antimicrobial peptides, antibiotic resistance, mechanism of action, bacterial protein targets

## Abstract

Antimicrobial peptides (AMPs) from amphibians represent a promising source of novel antibacterial agents due to their potent and broad-spectrum antimicrobial activity, which positions them as valid alternatives to conventional antibiotics. This review provides a comprehensive analysis of the mechanisms through which amphibian-derived AMPs exert their effects against bacterial pathogens. We focus on the identification of bacterial protein targets implicated in the action of these peptides and on biological processes altered by the effect of AMPs. By examining recent advances in countering multidrug-resistant bacteria through multi-omics approaches, we elucidate how AMPs interact with bacterial membranes, enter bacterial cells, and target a specific protein. We discuss the implications of these interactions in developing targeted therapies and overcoming antibiotic resistance (ABR). This review aims to integrate the current knowledge on AMPs’ mechanisms, identify gaps in our understanding, and propose future directions for research to harness amphibian AMPs in clinical applications.

## 1. Introduction

AMPs are naturally occurring molecules found in all living organisms, playing essential roles in innate immunity. These peptides, which are produced by various organisms, including amphibians, have gained considerable attention in recent years due to their potent antimicrobial properties. AMPs are able to target and kill a wide range of pathogens, including both Gram-positive and Gram-negative bacteria, and are especially promising in the context of combating antibiotic-resistant strains [1]. Unlike traditional antibiotics, which often target specific bacterial processes, AMPs typically interact with bacterial membranes, disrupting their integrity and leading to cell death. This broad mechanism of action allows AMPs to overcome some of the limitations of conventional antibiotics, such as the development of resistance.

Despite their potential, the precise mechanisms through which AMPs exert their antimicrobial effects are still not fully understood. While many AMPs have been shown to disrupt bacterial membranes, recent studies suggest that they may also target intracellular components, such as DNA, RNA, and proteins, further complicating their mode of action. This complexity presents both a challenge and an opportunity: understanding how AMPs interact with bacterial cells at a molecular level could pave the way for the development of new, more effective antimicrobial therapies. Identifying specific bacterial proteins targeted by AMPs is crucial, as it could lead to novel therapeutic strategies that enhance the ability to fight resistant infections.

In the ongoing fight against antibiotic resistance (ABR), researchers are intensively exploring novel antimicrobial agents to address the growing challenges posed by drug-resistant bacteria [2]. This effort is focused on developing innovative strategies that can effectively target and eradicate resilient bacterial strains. One promising approach is the elucidation of the mechanism of action (MOA) of antimicrobial molecules capable of overcoming ABR at the molecular level, with the aim to identify their specific protein targets within bacterial cells to develop innovative therapeutic interventions [3,4].

Among the many sources of AMPs, peptides derived from amphibian skin—particularly frog skin—are of particular interest. These peptides are notable for their broad-spectrum antimicrobial activity and their effectiveness against antibiotic-resistant bacteria [5].

The unique peptide compositions of amphibian skin secretions, which include a large proportion of cationic and hydrophobic residues, enable these peptides to interact effectively with bacterial membranes, which are typically negatively charged. The ability of these peptides to penetrate lipid bilayers and form pores in bacterial membranes is well documented [6]. They show high selectivity for the lipid bilayers of bacterial membranes, which typically have a high concentration of acidic phospholipids and low cholesterol content [7]. Additionally, many amphibian skin peptides contain specific structural features, such as α-helices or β-sheets, which are crucial for their ability to disrupt microbial membranes. The presence of disulfide bonds in some of these peptides provides stability and enhances their antimicrobial activity by maintaining their three-dimensional structures. Furthermore, amphibian skin peptides exhibit a remarkable ability to form pores or disrupt membrane integrity through various mechanisms, including the barrel-stave model, the carpet-like model, and the toroidal model [8].

However, recent research suggests that, at sublethal concentrations, amphibian-derived AMPs may induce bacterial death through mechanisms different from membrane disruption [9]. Recently reported scientific evidence suggests that these peptides can interact with intracellular protein targets, leading to cellular damage.

**Figure 1:**
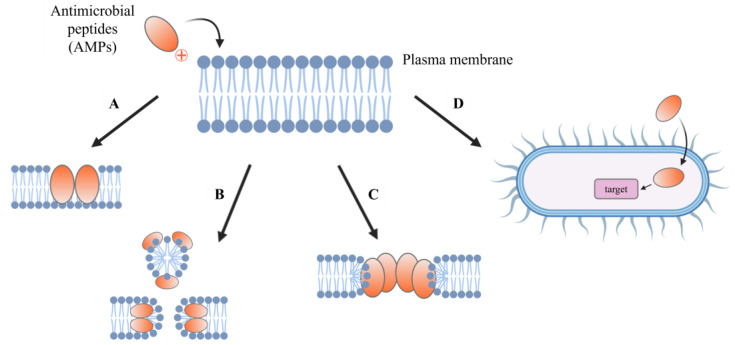
Mechanisms of action of antimicrobial peptides. (A) The barrel-stave model, where peptides form transmembrane pores. (B) The carpet model, where peptides cover the membrane surface and disrupt its integrity. (C) The toroidal model, where peptides induce curved pores in the membrane. (D) A representation of a non-lytic mechanism, where the peptide interacts with intracellular targets instead of disrupting the membrane.

All of the mechanisms described are illustrated in Figure 1.

This review describes biochemical and proteomic procedures, along with in silico modeling, that can offer valuable approaches for the investigation of the mechanisms of action of amphibian-derived AMPs, thereby enhancing our understanding of antimicrobial resistance and facilitating the development of innovative strategies through the identification of protein targets.

## 2. AMPs from Amphibians

### 2.1. Influence of Structural Properties of Amphibian Antimicrobial Peptides on Their Antimicrobial Activity

The antimicrobial activity of amphibian-derived AMPs is critically dependent on their structural properties, including the peptide length, net charge, and amino acid composition [10]. Most amphibian AMPs range from 10 to 30 amino acid residues in length, with some extending up to 50 residues [11], and are positively charged due to the presence of lysine, arginine, and histidine residues [12]. The short length of these peptides facilitates efficient membrane penetration, while their positive charge enhances their binding to the negatively charged phospholipids of pathogen membranes [13].

The structural diversity of AMPs, driven by variations in the amino acid composition, is essential to their adaptability and antimicrobial efficacy. This variety allows them to interact with a wide range of microbial membranes, often through mechanisms like membrane disruption [14], pore formation [15], or interference with intracellular targets [9]. Moreover, the analysis of the three-dimensional structures of these peptides has revealed that the spatial position of the amino acid residues plays a key role in their functional activity [16].

Among the most studied AMPs from amphibians, there are esculentins, magainins, maximins, ranacyclins, ranatuerins, temporins, and tigerinins [1], and their main features are reported in Table 1. These peptides exhibit broad-spectrum efficacy against a wide array of bacterial pathogens, encompassing both Gram-positive and Gram-negative species.

Esculentins, magainins, and temporins are intrinsically disordered in aqueous environments but adopt amphipathic α-helical conformations upon interaction with membranes or in the presence of organic solvent mixtures [26]. This structural transition is essential for their antimicrobial activity, as it enables the peptides to spontaneously cross the membrane and affect intracellular targets, thereby modulating their MOA [27].

**Figure 2:**
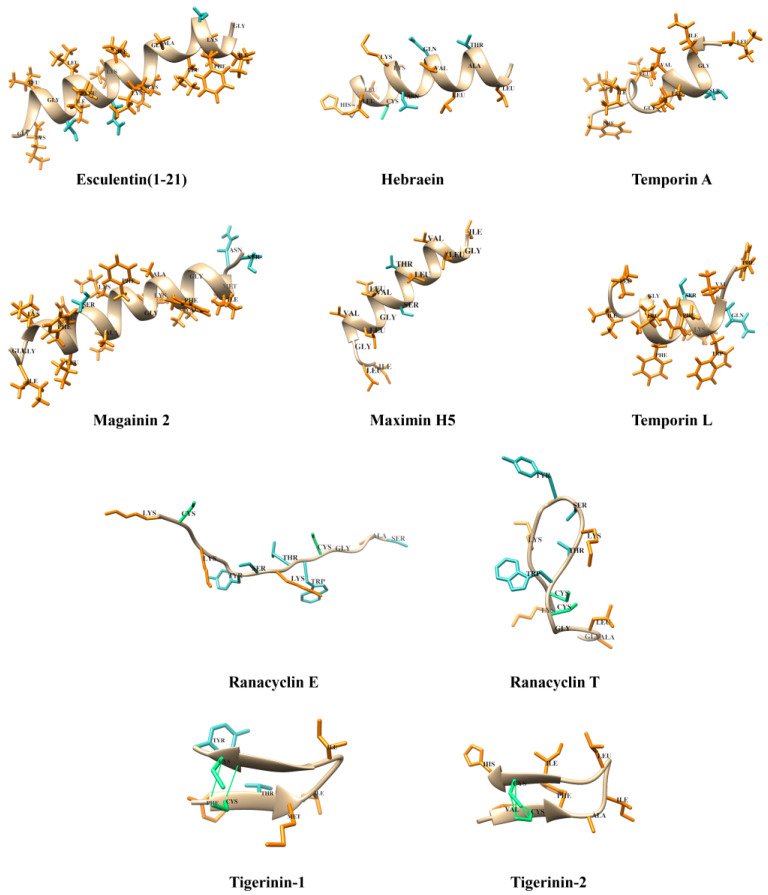
Some 3D models of amphibian-derived AMPs. Hydrophobic residues are orange, hydrophilic residues are blue, and disulfide bridges are green. The PDB codes are as follows: 5XDJ for Esculentin(1–21), 2MAG for Magainin-2, AF-P83663-F1-v4 for Ranacyclin-E, 2MMA for Temporin-A, 6gs5 for Temporin-L. All structures that do not have a PDB/AlphaFold code have been simulated using AlphaFold (https://alphafold.com/about, accessed on 10 November 2024).

The β-sheet and β-hairpin architectures of AMPs are also fundamental to their biological function. Peptides in the tigerinin family depend on the structural stability provided by disulfide bonds in these secondary structures to maintain functionality [28]. Similarly, members of the ORB family, such as ranacyclins, show a conserved structural element known as the ‘Rana box’, characterized by an intramolecular disulfide bridge at the C-terminal region. This bond stabilizes a macrocyclic arrangement composed of seven to nine amino acid residues. The highly conserved GCWTKSXXPKPC sequence within this region plays a central role in maintaining structural integrity, antimicrobial efficacy, and trypsin inhibition [29]. These structural features are described using 3D models, which illustrate key conformational changes and structural motifs (Figure 2).

Beyond the secondary structure, the peptide net charge exerts a significant influence on the antimicrobial spectra of amphibian AMPs. Positively charged AMPs, such as Magainin-2 from *Xenopus laevis*, exhibit broad-spectrum antibacterial activity, effective against both Gram-negative [18] and Gram-positive bacteria [30]. Conversely, Maximin-H5, an AMP derived from *Bombina maxima*, is anionic due to its lack of basic residues, limiting its antimicrobial activity primarily to Gram-positive species, such as *Staphylococcus aureus* [19,31]. Several other antimicrobial anionic peptides (AAMPs) with net charges ranging from −1 to −8 have been identified in frogs, toads, and salamanders, and most of these peptides exert antibacterial activity through interaction with the membrane [32]. In particular, they exhibit a pH-dependent mechanism of biological activity, which is relatively rare among amphibian AMPs [33].

Thus, the charge, sequence, molecular size, amphipathicity, hydrophobicity, and secondary structure constitute the six critical physicochemical and structural factors governing the bioactivity of amphibian-derived AMPs.

### 2.2. Advantages and Challenges of Amphibian-Derived AMPs in Antimicrobial Therapy

Amphibian-derived AMPs present several advantages in antimicrobial therapy due to their potent activity at low concentrations, which enhances their therapeutic potential while minimizing their cytotoxicity to host cells. These peptides typically exhibit low toxicity to human cells, making them promising candidates for the development of new antibacterial therapies [34]. This phenomenon arises because AMPs can selectively target microbial membranes at lower doses, minimizing adverse interactions with mammalian cells. At these sub-toxic concentrations, AMPs maintain their antimicrobial efficacy while reducing their cytotoxic effects on host cells. However, as their concentrations increase, the likelihood of AMP interactions with mammalian cell membranes also rises, leading to a higher risk of toxicity. Consequently, the therapeutic index of AMPs is often optimized within this lower concentration range to balance antimicrobial potency and safety. Additionally, their structural diversity and ability to modulate immune responses provide a versatile platform for therapeutic innovations [35,36,37,38].

Indeed, magainins, among the most well-known AMPs isolated from amphibian skin, have been investigated for applications in wound healing and against resistant bacterial infections due to their relatively low toxicity to mammalian cells [39].

Temporins, a family of small AMPs derived from the skin of *Rana temporaria*, have demonstrated efficacy against various bacterial strains, including Methicillin-resistant *Staphylococcus aureus* (MRSA) [40,41]. Their selectivity for bacterial membranes over mammalian cell membranes, coupled with minimal hemolytic activity, makes them promising candidates for therapeutic use [42].

Esculentin-1, a potent antimicrobial peptide from the skin of *Rana esculenta*, exhibits strong antibacterial activity, particularly against Gram-negative bacteria such as *Escherichia coli (E. coli)* and *Pseudomonas aeruginosa (P. aeruginosa)*. It also possesses anti-inflammatory properties and low toxicity to human red blood cells, enhancing its suitability for pharmaceutical applications [43].

Despite these advantages, several challenges must be addressed before these molecules might become available for the clinical application of AMPs. One major drawback is their poor stability in biological environments, where they are rapidly degraded by proteolytic enzymes like trypsin, serine, and cysteine proteases, reducing their bioavailability and therapeutic efficacy [44]. The positive charges of amino acids like arginine (Arg) or lysine (Lys) often render them susceptible to degradation by trypsin and serine proteases [45]. Cysteine proteases are known to cleave peptide bonds where amino acids like glycine, alanine, valine, phenylalanine, and tyrosine are present [46].

AMPs from amphibian skin, particularly those with multiple cysteine residues forming disulfide bonds, are more resistant to both cysteine and serine proteases compared to their linear counterparts. Amphibian-derived AMPs utilize these cysteine bonds to form stable conformations, such as beta-sheets or knotted structures, which prevent cleavage by proteolytic enzymes and help the peptides to remain active in environments with high protease concentrations. For example, esculentins from frog skin exhibit partial resistance due to the protective disulfide bonds, preserving critical regions for antimicrobial activity. This stability is particularly important for therapeutic applications, where AMPs may encounter high levels of proteolytic enzymes [47].

Furthermore, studies on de novo AMP design have suggested strategies to improve their resistance to protease degradation, such as using the D-enantiomers of peptides. The isomerization of amino acids in AMPs can enhance the resistance to enzymatic degradation and improve the antimicrobial effectiveness [48]. For example, converting L-amino acids to D-amino acids in the peptide Esculentin(1–21) to form Esculentin(1–21)-1c conferred greater resistance to proteolytic degradation and reduced cytotoxicity against eukaryotic cells, while also inhibiting *P. aeruginosa* biofilm formation and growth [48,49].

An alternative approach to address proteolytic degradation involves the development of small bifunctional peptides that combine both protease inhibition and antimicrobial activity. The flexible structural motifs of tigerinins—short, non-helical AMPs stabilized by a single disulfide bridge—have inspired the design of disulfide-bridged peptides. Research has shown that these disulfide bonds not only stabilize peptide structures but are also essential in maintaining the conformation necessary for target interaction, thereby enhancing the stability and overall antimicrobial potency of amphibian-derived AMPs [28,50].

**Figure 3:**
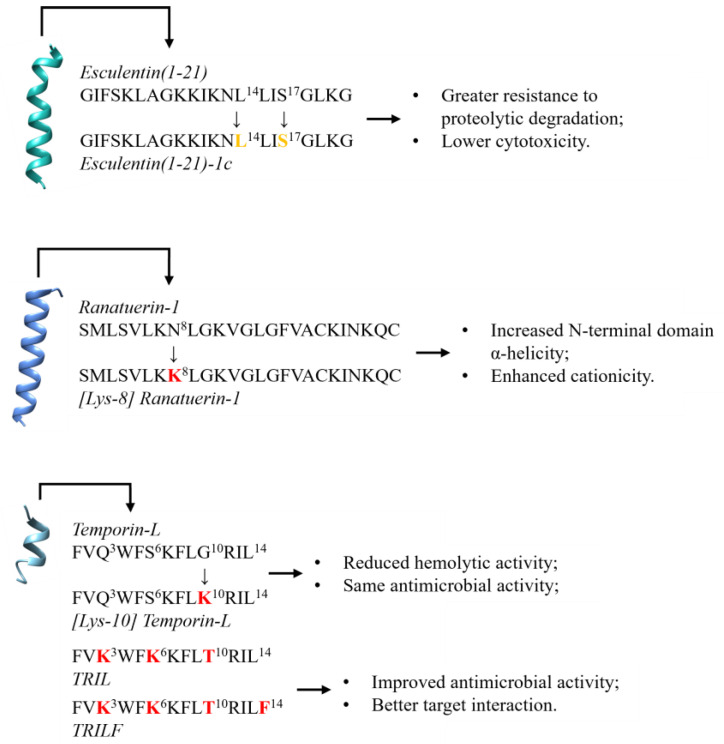
Analysis of the correlation between sequence modifications in well-characterized antimicrobial peptides—Esculentin(1–21) [67], Ranatuerin-1 [68,69], and Temporin-L [70]—and the functional advantages conferred by these alterations. Isomerizations are indicated in yellow, while amino acid substitutions are highlighted in red. The PDB and AlphaFold codes are as follows: 5XDJ for Esculentin-1a(1–21), AF-P82741-F1-v4 for Ranatuerin-1, and 6GS5 for Temporin-L.

Beyond protease-mediated cleavage, bacteria could potentially develop resistance to AMPs through several other adaptive mechanisms. One common strategy involves altering the charge and composition of the bacterial cell membrane. However, recent research has suggested the development of novel modified AMPs (Figure 3), which potentially enhance their antimicrobial efficacy and minimize side effects, bypassing this resistance mechanism [51]. For instance, substituting lysine (Lys) for asparagine (Asn) at position 8 in Ranatuerin-1 increased the positive charge and potency against both Gram-positive and Gram-negative bacteria, with only a minimal increase in hemolytic activity. The improved antimicrobial potency of [Lys-8] Ranatuerin-1 likely originates from the increased α-helicity of the N-terminal domain and enhanced cationic charge [52], which are critical for binding to negatively charged phospholipids in bacterial cell membranes [53,54].

Recent research on Temporin-L analogs has identified a similar enhancement in activity. Temporin-L is the most effective antimicrobial peptide but is toxic to human erythrocytes, which makes it essential to create synthetic analogs with a greater therapeutic index. Over time, Mangoni et al. have examined the structure–activity correlations of a collection of Temporin-L derivatives, focusing on the correlation between the peptide’s α-helix content cationic residue type and its antibacterial properties. Their study established that the position and type of positively charged residues significantly impact the peptide’s functions. For instance, replacing the guanidinium group at position 11 (Arg) with an amine (Lys) successfully reduced the hemolytic activity while maintaining the antimicrobial properties [55]. The guanidinium group on arginine is highly charged and capable of forming multiple hydrogen bonds, which enhances its interaction with cell membranes. While this feature is beneficial in targeting bacterial cells, it can also lead to the disruption of human cell membranes, including red blood cells, resulting in hemolysis. Lysine, on the other hand, carries a simpler amine group with a lower charge density, which reduces its ability to be inserted deeply into lipid bilayers, thereby limiting its disruptive impact on eukaryotic cells. This highlights a promising direction for the development of more selective temporin-based anti-infective agents, underlining the complex relationship between the peptide’s structure and biological function.

In this regard, Temporin-L has been shown to inhibit the FtsZ protein, a key component in bacterial cell division [56,57]. Building on this discovery, two analogs of Temporin-L, named TRIL and TRILF, were designed with the aim of enhancing the FtsZ inhibition [58]. These findings provide a foundation for the development of novel drugs specifically engineered to target FtsZ and disrupt cell division [59].

Moreover, AMPs may also trigger host immune responses, leading to potential immunogenicity concerns [60]. One potential alternative to address these challenges and improve the pharmacokinetics and therapeutic performance of AMPs is the development of drug delivery systems that encapsulate and protect them from degradation. By incorporating peptides into advanced delivery systems, such as nanoparticles [61,62,63] or liposomes, it is possible to enhance their stability and reduce their immunogenicity [64,65,66].

## 3. Amphibian-Derived Antimicrobial Peptides Targeting Essential Bacterial Proteins

Investigation into the mechanisms underlying the antimicrobial activity of promising amphibian-derived AMPs may facilitate the identification of novel therapeutic agents. This is closely associated with the identification of the specific targets within bacterial systems. Bacteria possess sophisticated defense mechanisms and have several critical targets that are essential for their biological functions [71]. The following section provides a detailed discussion of the primary bacterial targets that have been identified through the study of the mechanisms of action of various amphibian-derived AMPs by genomic and proteomic strategies.

### 3.1. Targeting Bacterial Outer Membrane Proteins

Amphibian-derived AMPs have been shown to directly interact with the essential components of the outer membrane (OM), typical of Gram-negative bacteria, interfering with the main functions of this highly structured barrier [72]. One of the critical components of the OM is lipopolysaccharides (LPS), which stabilize the membrane and contribute to the immune evasion capabilities of the bacteria, hindering the penetration of antibiotics.

Chen et al. reported that some AMPs belonging to temporins, brevinins, and esculentins can interact with LPS molecules, disrupting their structural integrity and causing membrane destabilization [29]. This leads to increased permeability, which compromises the bacterial membrane’s selective barrier function, allowing the entry of toxic compounds and ultimately causing cell lysis.

However, beyond targeting the lipid components, some amphibian-derived AMPs are also known to interact with specific OM protein targets. Outer membrane proteins (OMPs), for instance, are proteins that form channels through which small molecules and ions pass in and out of the bacterial cell [73]. They are involved in multiple critical cellular functions in Gram-negative bacteria, including nutrient uptake, protein secretion, and virulence-related adhesion.

In *E. coli*, following synthesis in the cytoplasm, OMPs undergo translocation across the inner membrane via the SecYEG translocon in a SecA-dependent manner within the periplasm. The Skp protein and the SurA chaperon drive nascent OMPs into the β-barrel assembly machinery (BAM) complex [74], a heterooligomer with a mass of ~200 kDa, composed of five proteins designated as BamA-BamE [75].

**Figure 4:**
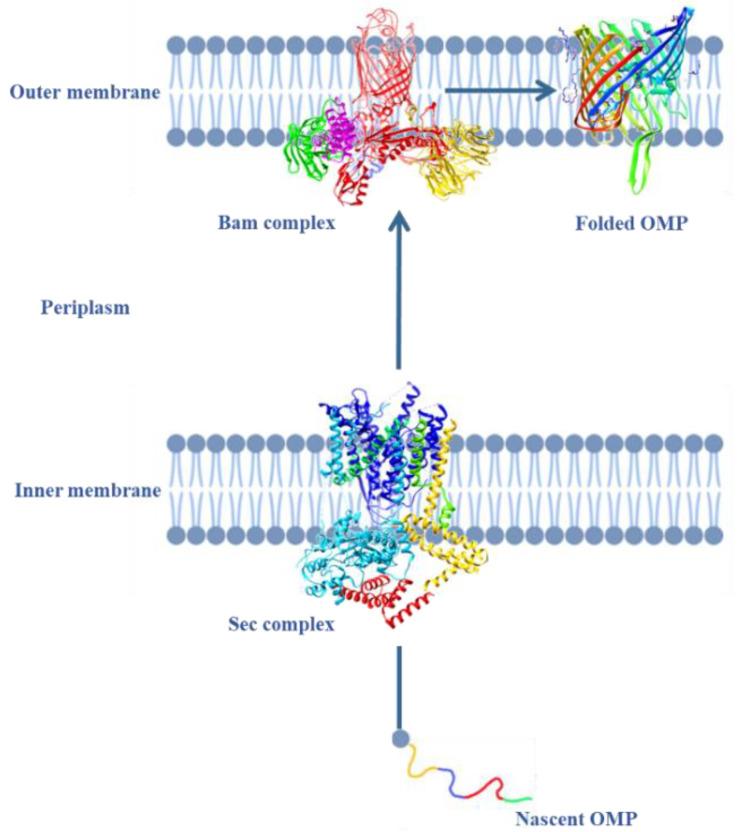
Representation of OMP folding. OMPs fold in the periplasm by binding to POTRA domains in a closed β-hairpin shape, connecting the two terminals and facilitating folding. This weakens the connection between the β1 and β16 strands of the BamA β-barrel, allowing OMPs to exit through the lateral opening and integrate into the OM. The structures were modeled using CHIMERA. The PDB codes are as follows: 7AFT for Sec complex, 5JLO for Bam complex, and 4C4V for folded OMP.

Among these subunits, BamA plays a crucial role in the folding and insertion of nascent β-barrel OMPs in the OM (Figure 4) and might then represent, together with OMPs, a significant antibiotic target in Gram-negative bacteria.

Amphibian-derived AMPs have been observed to block OMPs, disrupting essential nutrient and ion transport processes, which in turn leads to metabolic stress and bacterial death. In a study on *E. coli*, Esculentin-1b(1–18) [Esc(1–18)], an amphibian-derived antimicrobial peptide, was shown to significantly impact the expression levels of OMPs. Proteomics analysis revealed the marked downregulation of key OMPs, including OmpC, OmpF, and NmpC, which are responsible for the passive diffusion of small hydrophilic molecules across the bacterial OM [76].

AMPs have also been shown to hinder the function of the Bam complex, thereby impairing the assembly of essential proteins required in maintaining the integrity and function of the OM. This interference further weakens the bacterial defense system, making the organism more susceptible to environmental stress and immune responses.

Recent functional proteomics experiments in *E. coli* identified the Bam complex (ABCD) as the main target of Magainin-2 (Mag-2) a well-known antimicrobial peptide isolated from the African clawed frog *Xenopus laevis*. Mag-2 can specifically bind BamA within the large cavity of the β–barrel structure, impairing the proper folding and allocation of OMPs [77].

**Figure 5:**
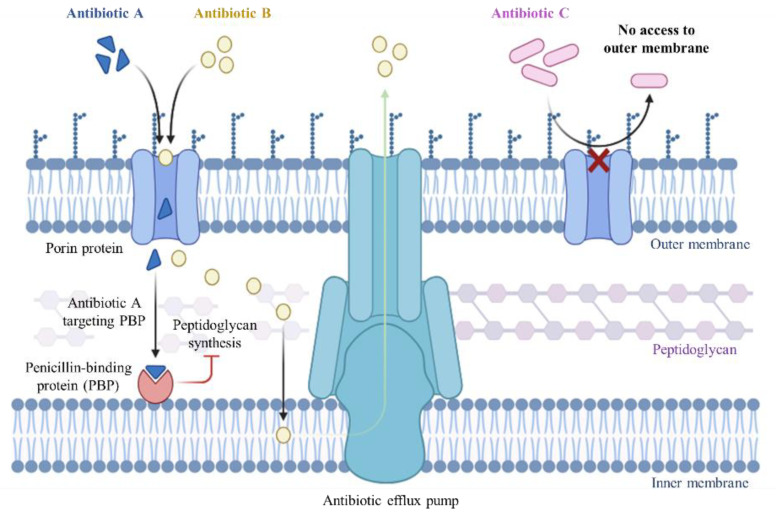
Bacterial efflux pumps in Gram-negative bacteria.

Besides OMPs, amphibian-derived AMPs can also target specific components of efflux pumps, a multiprotein complex constituting an integral component of microbial defense mechanisms against antimicrobial agents. These membrane-spanning proteins are involved in the active extrusion of a diverse array of substrates, including antibiotics, toxins, and other xenobiotics, from bacterial cells, thereby conferring a multidrug resistance phenotype to bacteria [78] (Figure 5).

The recent identification of the diastereomer Esc(1–21)-1c [79], along with transcriptional and differential proteomic analysis, has revealed the inhibition of the MexAB-OprM efflux pump activity, providing clear evidence of a mechanism responsible for the increased susceptibility of *P. aeruginosa* to various antibiotics in combination with Esc(1–21)-1c [49].

### 3.2. Inhibition of Energy Metabolism

AMPs derived from amphibians have emerged as potent inhibitors of energy metabolism in bacteria, disrupting key processes involved in energy generation, primarily through the targeting of bacterial membranes and enzymes essential for ATP synthesis. The disruption of energy metabolism compromises the bacterial cell’s ability to generate ATP, leading to a critical energy deficit. As a result, fundamental cellular activities are impaired, including membrane transport, protein synthesis, motility, and biofilm formation, which are vital for bacterial survival and pathogenicity. Furthermore, the depletion of energy reserves hinders the bacteria’s ability to mount adaptive responses, such as resistance mechanisms, making them more vulnerable to immune clearance and antimicrobial agents. This mode of action limits the development of resistance and broadens the therapeutic potential of AMPs against multidrug-resistant pathogens. Thus, AMPs targeting energy metabolism represent a promising frontier in the development of next-generation antimicrobials.

Magainin-1, a well-characterized AMP, has been shown to inhibit energy metabolism proteins in *E. coli* in a comparative transcriptomics analysis of susceptible and resistant strains. An RNA-seq analysis revealed the differentially expressed genes (DEGs) involved in bacterial metabolism, particularly energy-related processes. In susceptible strains treated with Magainin-1, the upregulation of genes related to energy metabolism and sulfate transport was observed, suggesting that the peptide significantly disrupts metabolic pathways to maintain energy homeostasis under antimicrobial-induced stress conditions [80].

Moreover, amphibian-derived AMPs have been shown to exert variable inhibitory effects on ATP synthase, a key enzyme responsible for cellular energy production through oxidative phosphorylation.

**Figure 6:**
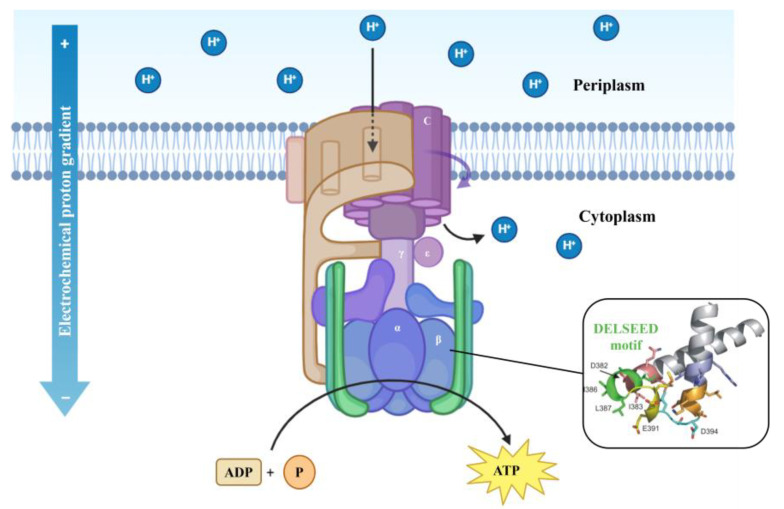
Schematic representation of the ATP synthase complex in bacteria. The structure of the DELSEED motif is from Mnatsakanyan et al. [81].

In a study evaluating the inhibition of ATP synthase from *E. coli*, several peptides, including ascaphin-8, aurein-2.2, aurein-2.3, carein-1.9, citropin-1.1, dermaseptin, maculatin-1.1, magainin II–amide, melittin-related peptide (MRP), and its amide form, as well as melittin and melittin–amide, demonstrated significant activity. Among these, MRP–amide was identified as the most potent inhibitor, achieving nearly complete inhibition (∼96%), with an IC50 of approximately 3.25 µM. The inhibitory effects of these peptides are attributed to their amphipathic α-helical structures, which facilitate interaction with the negatively charged DELSEED motif [81] on the β-subunit of ATP synthase (Figure 6). The presence of an amide group at the C-terminal further enhances the inhibitory potential, as observed in melittin–amide and MRP–amide, which both exhibited stronger inhibition compared to their non-amidated counterparts. These findings suggest that amphibian-derived peptides can serve as potent modulators of ATP synthase activity, potentially linking their antimicrobial properties to the disruption of ATP synthesis in target cells.

### 3.3. Impact on Cell Division

**Figure 7:**
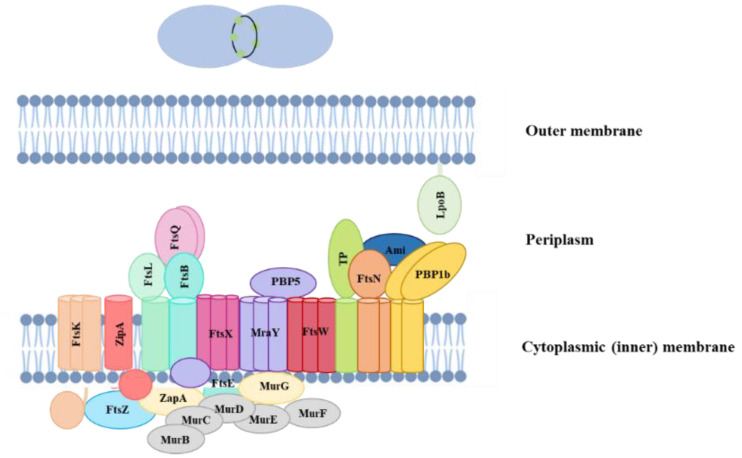
Schematic representation of the proteins involved in the process of cell division, constituting the divisome complex.

One of the most studied vital targets in bacteria is the machinery governing cell division, which plays a pivotal role in their survival and proliferation. The process of bacterial cell division, characterized by binary fission, is regulated by the highly coordinated interplay of several proteins (Figure 7) [82], and targeting the essential proteins responsible for bacterial cell division can lead to cell death and hinder the ability of bacteria to propagate. Moreover, proteins involved in bacterial cell division often do not have a counterpart in eukaryotic cells, rendering them promising candidates for antimicrobial intervention [83].

Among the myriad proteins orchestrating cell division, FtsZ assumes a pivotal role as the primary cytomotor organizer of the divisome in the majority of bacteria [84]. FtsZ is a tubulin-like GTPase belonging to the divisome complex that assembles into polar protofilaments in which the GTP-binding site of one monomer is at the binding interface with the next monomer. The assembly process is regulated by several accessory proteins [85], and FtsZ polymers form a dynamic ring-like structure, known as the Z-ring, at the future site of cell division. The Z-ring formation represents the earliest known step in bacterial cytokinesis and recruits a set of other cell division proteins essential for the function of dividing the cell [57]. Given its central function in divisome assembly, FtsZ becomes a crucial target for interventions aimed at modulating or disrupting the cell division process. Strategies directed towards FtsZ may hold significant promise in developing approaches to control bacterial growth and proliferation.

An investigation of the mechanism of action of Temporin-L on *E. coli* cells by functional proteomic approaches identified a number of putative interactor proteins belonging to the divisome machinery. In silico docking prediction, biochemical experiments, and biophysical techniques using the recombinant protein demonstrated that the GTPase FtsZ was the specific target of Temporin-L, revealing a competitive inhibition mechanism [56]. Spectroscopic measurements demonstrated that, upon incubation with the peptide, bacterial cells were unable to divide, forming long necklace-like cell filaments, indicating that Temporin-L inhibit FtsZ, impairing cell division [56].

## 4. Discussion and Future Considerations

Amphibian-derived AMPs represent a promising avenue for the development of new therapeutic agents, especially in the face of rising antibiotic resistance. These peptides exhibit a variety of MOAs, from membrane disruption to interference with essential bacterial processes and to specific protein targeting, bypassing common resistance mechanisms, such as efflux pump activation, enzymatic degradation, and modifications of target sites. However, challenges such as the peptides’ stability in biological environments and their potential cytotoxicity must be addressed.

This review discussed the recent mechanisms by which intracellular protein targets are inhibited by the most common amphibian-derived AMPs and their derivatives. These findings suggest that multi-omics approaches are crucial in uncovering additional AMP mechanisms of action and optimizing their application in next-generation antimicrobial therapies. One notable group of protein targets of AMPs is located in the outer membrane (OM) in both Gram-positive and Gram-negative bacteria. The inhibition of the proper folding and/or localization of outer membrane proteins (OMPs) by AMPs compromises bacterial membranes’ integrity, impairing essential processes such as nutrient transport and structural maintenance, ultimately leading to bacterial cell death. Moreover, the negative effect exerted by AMPs on bacterial efflux pumps might pave the way for the development of alternative therapeutic strategies based on the synergy of AMPs and antibiotic formulations, re-evaluating the efficacy of conventional antibacterial agents.

Other proteins demonstrated to be specifically targeted by AMPs are involved in energy metabolism or belong to complex systems essential for bacterial survival. AMPs can induce a critical energy deficit in bacterial cells, impairing vital processes such as motility, biofilm formation, and immune evasion. This approach could also limit bacterial adaptation and resistance development, providing a significant therapeutic advantage. Targeting bacterial vital processes such as cell division machinery further enhances the therapeutic potential of AMPs. Proteins involved in bacterial cell division are critical for reproduction and survival and, due to their absence in human cells, represent selective targets that minimize the collateral damage to host tissue, making them attractive candidates for novel antimicrobial strategies.

The implications of these findings are significant, highlighting how the continued exploration of amphibian-derived AMPs and their mechanisms of action offers a valuable opportunity to develop targeted therapies that not only bolster our defenses against bacterial infections but also preemptively counter emerging resistance mechanisms. To date, innovative treatments based on amphibian-derived AMPs have been developed to address the growing challenge of antibiotic resistance. One notable example is Pexiganan, a synthetic peptide modeled after the magainin AMP found in frog skin, which has been formulated as a topical cream for the treatment of diabetic foot infections [86]. However, most peptides approved for clinical use come from bacteria (such as bacitracin and polymyxins) or are chemically synthesized to mimic the natural characteristics of antimicrobial peptides [87,88]. Amphibian peptides, on the other hand, are still the subject of preclinical and clinical studies to better understand how they can be modified or stabilized for therapeutic use in humans.

However, the AMPs discussed in this review have several important limitations that may hinder their widespread therapeutic application. One key limitation is the potential toxicity of AMPs to mammalian cells. While they are often more selective towards microbial cells than traditional antibiotics, high concentrations may still cause damage to host cells, leading to adverse side effects [89]. The interaction of AMPs with mammalian cell membranes can result in cytotoxicity, particularly when the peptides lack the specificity required for selective action. To mitigate this, ongoing research is focused on engineering more selective AMPs or developing delivery systems that target the peptides specifically to infected tissue, thus reducing the potential damage to healthy cells.

The clinical application of AMPs is also hindered by challenges such as instability, degradation, and low bioavailability in biological environments [90]. To address these issues, several innovative delivery strategies are being developed. One of the most promising approaches involves the use of nanoparticles, including lipid, polymeric, and metallic nanoparticles, which can encapsulate AMPs, protecting them from enzymatic degradation and improving their stability [91]. In addition, nanoparticles also promote more effective distribution and increased cellular uptake, thus enhancing their antimicrobial activity and bioavailability. Another interesting approach is the use of liposomes, lipid bilayer structures that encapsulate AMPs, preventing degradation and improving their stability in the bloodstream [92]. These systems can also be designed for targeted release by modifying the liposome surface with ligands or antibodies that direct the treatment to specific pathogens or tissue types. Moreover, the conjugation of AMPs with other compounds, such as polymers (e.g., PEGylation) or targeting peptides, can significantly improve their pharmacokinetic properties [93]. Conjugation with biodegradable polymers increases the solubility of AMPs, extends their duration of action, and prevents rapid renal clearance. Additionally, conjugation with molecules that enhance cellular uptake or provide a targeting mechanism helps to increase the therapeutic efficacy while reducing the side effects.

Recently, protein-based delivery systems that mimic the architecture of secretory granules of the endocrine system have been proposed to slowly release recombinant AMPs [62]. These newly synthesized secreting protein granules were shown to actively protect zebra fish from infection by the multi-resistant bacterium *Stenotrophomonas maltophila* [61].

In conclusion, although the translation of amphibian-derived AMPs into therapeutic agents presents a multifaceted challenge, involving several critical stages encompassing drug development, regulatory processes, and formulation strategies, their great potential provides a foundation for future research. This research should focus on improving their pharmacokinetic properties while addressing common limitations, such as reduced stability and increased toxicity. Structural modifications, the rational design of peptide analogs, and the development of protective drug delivery systems (e.g., nanoparticles and liposomes) may enhance the efficacy of AMPs against specific microbial targets while optimizing their pharmacological profiles for human therapeutics.

## Figures and Tables

**Table 1:** Classification and characteristics of amphibian-derived AMPs: sequence, structure, charge, and antibacterial activity.

Peptide	Sequence	Structure	Charge	Activity	Ref.
Esculentin(1–21)	GIFSKLAGKKIKNLLISGLKG	Helix	+6	Gram (−)	[17]
Magainin-2	GIGKFLHSAKKFGKAFVGEIMNS	Helix	+4	Gram (−) Gram (+)	[18]
Maximin-H5	ILGPVLGLVSDTLDDVLGI	Helix	−3	Gram (−)	[19]
Hebraein	HLELCKKNDQVLATELE	Helix	−4	Gram (−)	[20]
Ranacyclin-T	GALRGCWTKSYPPKPCK	Random	+5	Gram (−) Gram (+)	[21]
Ranacyclin-E	SAPRGCWTKSYPPKPCK	Random	+5	Gram (+)	[21]
Temporin-A	FLPLIGRVLSGIL	Helix	+2	Gram (+)	[22]
Temporin-H	LSPNLLKSLL	Helix	+1	Gram (+)	[23]
Temporin-L	FVQWFSKFLGRIL	Helix	+3	Gram (+)	[24]
Tigerinin-1	FCTMIPIPRCY	Bridge	+2	Gram (−) Gram (+)	[25]
Tigerinin-2	RVCFAIPLPICH	Bridge	+1	Gram (−) Gram (+)	[25]

## Data Availability

No new data were created or analyzed in this study.
